# The Role of Topical Tacrolimus in the Management of Inflammatory Bowel Disease: A Comprehensive Review

**DOI:** 10.3390/jcm13185518

**Published:** 2024-09-18

**Authors:** Danial Khayatan, Daniel A. Lemberg, Andrew S. Day

**Affiliations:** 1 Department of Dermatology, Columbia University Irving Medical Center, New York, NY 10032, USA; dk3364@cumc.columbia.edu; 2 Paediatric Gastroenterology, Sydney Children’s Hospital, Randwick, Sydney 2031, Australia; daniel.lemberg@health.nsw.gov.au; 3 Department of Paediatrics, University of Otago Christchurch, Christchurch 8011, New Zealand

**Keywords:** calcineurin inhibitor, tacrolimus, inflammatory bowel disease (IBD), therapeutics, inflammation, ulcerative colitis, Crohn’s disease

## Abstract

Management of ulcerative colitis and Crohn’s disease, the main subtypes of inflammatory bowel disease (IBD), focuses on the induction and maintenance of remission. Tacrolimus, a member of a group of drugs termed calcineurin inhibitors, may have a role in the medical management of IBD when given either systemically or topically. This review aimed to evaluate the available data focusing on the use of topical tacrolimus in the management of IBD. Reports of the use of topical tacrolimus in IBD were extracted from databases up to 31 May 2024. Topical tacrolimus therapy appears to have reasonable efficacy in the induction and maintenance of remission in patients with refractory IBD, with an acceptable safety profile. Overall, the available data are supportive of the use of topical tacrolimus in selected patients. Further comparative clinical studies are required to more fully delineate the role of this drug.

## 1. Introduction

The term inflammatory bowel disease (IBD) refers to a group of chronic immune-mediated disorders that affect the gastrointestinal (GI) tract [1]. While the exact cause of IBD is unknown, it is believed to result from a combination of genetic, environmental, and immunological factors [2].

The two main types of IBD are Crohn’s disease (CD) and ulcerative colitis (UC). While both feature acute and chronic inflammation, they are typically differentiated on the basis of the pattern and location of the inflammatory changes. CD is characterised by inflammatory changes in any location along the GI tract, from the mouth to the anus. On the other hand, the inflammatory changes seen in UC typically begin in the rectum and extend for a variable distance around the colon [3]. Further, both can lead to GI symptoms, such as pain or diarrhoea, along with more systemic concerns, such as weight loss or anaemia. In addition, the inflammatory changes seen in IBD can be complicated by the development of strictures (leading to obstruction), fistulae (with consequent perforation), and colitis-associated carcinoma (with associated mortality) [4]. Altogether, IBD can impact adversely upon quality of life and generate greatly increased healthcare costs [5].

IBD occurs more commonly in developed countries and is estimated to affect around 1.3% of the population in the United States [6,7]. Overall, the prevalence of IBD is increasing worldwide, particularly in developing countries over the last two decades [8,9]. While IBD can present at any age, many individuals are diagnosed in their late adolescence or early adult years [10,11].

The management of IBD involves a multidisciplinary approach that includes lifestyle modifications, nutritional interventions, pharmacotherapy, and surgery, depending on the type, severity, and location of the disease. The overall goal of management is to induce and maintain remission, achieve mucosal healing, prevent complications, and improve quality of life [12]. Although there are various drugs available to induce and/or maintain remission, none are curative.

Tacrolimus (TAC), a calcineurin inhibitor (CNI), has been shown to be efficacious as an immunosuppressive agent in individuals with IBD, especially those who have not had a suitable response to, or are intolerant of, a conventional therapy [13]. However, systemic use of TAC may be complicated by side effects [14]. These systemic side effects have led researchers and clinicians to explore topical delivery of TAC as a potential alternative. Topical administration could potentially provide localised immunosuppressive effects while minimising systemic exposure and associated adverse events [15]. Treating distal colonic disease with topically administered local therapies can effectively target inflammation locally, and minimise systemic drug exposure [16,17]. However, these agents are often underused due to patient preferences and healthcare provider bias or unfamiliarity. Topical therapies can be used alone for distal disease or combined with oral therapies for more extensive disease and may be discontinued after achieving a satisfactory clinical response or remission [18]. This report aims to review the current evidence on the use of topical TAC in the management of IBD, with a focus on understanding the rationale behind this route of administration. The report considers the efficacy of topical TAC, along with the safety profile, compared to systemic use and its potential role in clinical practice, particularly for patients who may benefit from localised treatment or those at higher risk of systemic side effects.

## 2. Methodology

Data were collected from Scopus, Google Scholar, PubMed, and Cochrane Library for in vitro, in vivo, and clinical studies published in English between 18 November 1991 and 31 May 2024. Search terms included “Inflammatory Bowel Disease” OR “IBD” AND “Tacrolimus” OR “FK(506)” OR “FK-506” OR “FK506” AND “Ulcerative Colitis” OR “UC” OR “Crohn’s disease” OR “Crohn’s disease” OR “CD” AND “Immunosuppressive” AND “Topical”.

## 3. Pre-Clinical Evaluations of Tacrolimus: Building the Case for Efficacy in Inflammatory Bowel Disease

TAC is a member of a family of drugs known as calcineurin inhibitors that inhibit calcium- and calmodulin-dependent phosphatases. Other members include cyclosporine and pimecrolimus. TAC binds FK506-binding proteins (FKBPs) [12]. Inhibition of the phosphatase activity and suppression of interleukin (IL)-2 transcription is caused by an impaired nuclear factor of activated T-cells (NFAT) translocation, which regulates IL-2 transcription and T-cell activation [19]. TAC inhibits phosphatase activity when it binds to calcineurin in a complex with immunophilin. Phosphatase inhibition results in reduced production of cytokines, such as IL-2, which suppresses the proliferation of T-cells [20].

TAC has been demonstrated to be effective in preventing graft-versus-host disease after bone-marrow and rejection after solid-organ transplantation [21,22]. Furthermore, TAC is used to treat autoimmune conditions such as autoimmune enteropathy [23]. TAC has a low bioavailability of around 30%, consequent to active intestinal excretion facilitated by proteins like ABCB1 (P-glycoprotein) and enzymes like CYP3A. ABCB1 contributes to efflux from enterocytes, while CYP3A primarily engages in substantial first-pass metabolism [24,25]. It is believed that most (up to 80%) of the absorbed TAC binds to its receptor FKBP-12, which is highly concentrated in erythrocytes, while about 15% binds to plasma proteins. The remaining fraction of free TAC is minimal, approximately 0.5% [26,27].

In vitro and in vivo studies have shown the anti-inflammatory properties of TAC. For example, Aomatsu and colleagues [28] investigated the effect of TAC on cytokine and chemokine production by human colonic myofibroblasts. TAC (1 μM) suppressed tumour necrosis factor (TNF)-α-induced human monocyte chemoattractant protein-1 (CCL2) and C-X-C motif chemokine ligand (CXCL)10 mRNA expression but not IL-6 or CXCL8. TAC inhibited CCL2 and CXCL10 expression dose-dependently, with effects at concentrations as low as 0.5 μM. The mechanism involved inhibition of p38 mitogen-activated protein kinase (MAPK) phosphorylation without significant effect on signal-transducer and activator-of-transcription (STAT)-1 phosphorylation. These findings demonstrate the specific anti-inflammatory actions of TAC at the cellular level.

Van Lierop et al. [29] studied the effect of TAC on 2,4,6-trinitrobenzene sulfonic acid (TNBS)-induced colitis in wild-type and Rag2-deficient mice. In wild-type mice, TAC reduced colitis development and activated T-cells, leading to decreased neutrophil recruitment in the colon. TAC inhibited production of CXCL1, CXCL2, and CXCL5. Rag2-deficient mice showed a moderate increase in lamina propria neutrophils without TAC treatment. The study suggests TAC suppresses colitis by inhibiting T-cell activation and subsequent T-cell-mediated neutrophil recruitment.

## 4. Topical Application of Tacrolimus in Other Conditions

Topical TAC has been evaluated and shown to be efficacious in several skin diseases. For example, Schauber et al. [30] explored the potential of topical TAC in the management of perianal eczema. TAC ointment (0.1%) was applied twice daily for two weeks to a group of 24 people. All returning patients exhibited clinical improvement, evaluated through macroscopic appearance and clinical scores. This short-term trial underscores the safety, efficacy, and well-tolerated nature of topical TAC for perianal eczema.

Ucak et al. [31] assessed the efficacy of topical TAC in patients with atopic dermatitis (AD) experiencing perianal itching (PA). Half the group of 32 patients were treated with 0.03% TAC ointment, and the rest were treated with Vaseline (as a placebo). Both groups applied their respective treatments twice daily for four weeks and then switched treatments after a two-week washout period. The findings indicated a notable reduction in disease severity, quality-of-life impact and itching intensity in the active group at weeks 4 and 6 of treatment (*p* < 0.05). This study established that topical TAC treatment was well-tolerated, and effectively managed persistent PA in patients with AD. Epidermolytic acanthoma, a rare and typically benign anogenital tumour, is characterised by histological features like epidermolytic hyperkeratosis [32]. While most cases are asymptomatic, some are bothered by a troublesome itch. In a single case report, a 55-year-old man with symptomatic anogenital epidermolytic acanthomas, refractory to other interventions, was treated with topical 0.1% TAC ointment twice daily for two weeks with a significant reduction in itch [32].

## 5. Topical Tacrolimus for the Management of Perianal or Cutaneous Manifestations of IBD

Nanaeva et al. [33] conducted a prospective randomised trial involving 20 patients with perianal CD, specifically anal fissures and rectal fistulas. The study group (*n* = 11) received systemic therapy with azathioprine, along with TAC ointment, topically. In contrast, the control group (*n* = 9) received a different systemic therapy, along with hormone (oestrogen) ointment and metronidazole suppositories. After six weeks, fissure healing was observed in five of the study group and three of the control group. At 12 weeks, fissure healing and fistula closure were noted in six of the study group and three of the control group, with a reduction in the perianal CD activity index in the study group. These results suggest that 0.1% TAC ointment is an effective treatment for patients with perianal CD, leading to improved outcomes and reduced disease activity compared to alternative therapies.

Hart et al. [34] evaluated TAC ointment in 19 patients with refractory perianal CD in a 12-week placebo-controlled RCT. While three of the four patients with ulcerating disease had a clinical response to TAC, no benefits were seen in the placebo group. However, TAC ointment did not benefit any of the individuals with fistulizing disease. Two of these subjects developed a perianal abscess despite TAC treatment. The side-effect profile was favourable overall, and TAC was detectable on trough levels in just two instances.

A number of reports have also indicated that topical administration of TAC as an ointment may have a role in the management of specific cutaneous manifestations of IBD (Table 1). In a study conducted by Casson et al. [35], topical TAC was evaluated in a group of children with treatment-resistant perioral (*n* = 3) or perineal CD (*n* = 6). Substantial improvements were observed in seven out of the eight children after six weeks of treatment with 0.5 mg/g ointment, with sustained complete healing within a variable period spanning 1 to 6 months. Intriguingly, two initially responsive patients experienced disease exacerbation upon discontinuation or rapid dose reduction of TAC, leading to eventual proctectomy. This led to a strategic shift to a more gradual tapering approach for drug concentration, resulting in successful outcomes for six children. Among them, four received intermittent treatment, while two were maintained on reduced regular dosages (ranging from 0.1 to 0.3 mg/g), with follow-up periods extending up to 3.5 years. Crucially, serum levels of TAC were consistently undetectable in all patients, indicating minimal systemic absorption.

In another study, Rice et al. [36] investigated the efficacy of topical TAC in 20 adults with various cutaneous manifestations of CD. While most of the subjects had perineal or perioral CD, others had metastatic CD or pyoderma gangrenosum. All of the subjects were treated with topical TAC 0.1% ointment once daily for 12 weeks, up to a maximum total dose of 90 g. Those who relapsed subsequent to the completion of the initial course could continue TAC treatment over 12 months. Fifteen of seventeen patients who completed their initial course had improvements based on a specific physician’s global severity scale. In this case series, the long-term application of 0.1% TAC to affected skin and mucosal areas was well tolerated and safe, with undetectable serum levels. Overall, this approach proved effective in addressing various cutaneous manifestations of CD.

Shah et al. [37] presented a case of a 22-year-old woman with CD who had a discharging orofacial lesion on the right side of her face. The lesion had not responded to various prior treatments. Twice daily application of TAC 0.1% ointment resulted in a gradual reduction in lesion size, minimal discharge, and improved oral features over 12 months.

## 6. Per-Rectal Administration of Tacrolimus (Ointment, Suppository, or Enema)

TAC has shown promise in treating inflammatory bowel disease, particularly distal ulcerative colitis and refractory proctitis (Table 2). Lawrence et al. [25] conducted a pilot study using rectal TAC ointment (0.3 mg/mL) for 8 weeks in patients with refractory proctitis. Six of the eight patients achieved remission, allowing corticosteroid reduction in five. Building on these findings, Van Dieren et al. [38] expanded the investigation to 19 patients with refractory left-sided colitis or proctitis, using TAC as an enema or suppository. Their results corroborated the earlier study, with 13 patients showing improved disease activity after 4 weeks and maintaining low serum trough levels (tacrolimus trough levels < 3.2 μg/L). Further validating these outcomes, an Australian randomised controlled trial compared rectal TAC (0.5 mg/mL) to a placebo in 21 adults over an 8-week trial [39]. The study demonstrated significantly higher clinical response rates with TAC (73% vs. 10%, *p* = 0.004), as well as improved remission and mucosal healing rates.

These promising results led to a larger, more comprehensive study conducted in the Netherlands and Belgium [40]. This randomised, controlled, double-blind trial compared TAC suppositories (2 mg) with beclomethasone (3 mg) in 85 patients over 4 weeks. Both treatments showed comparable efficacy, with 63% of patients treated with TAC achieving clinical response. Exploring alternative formulations, an open-label study evaluated a simple TAC enema preparation (1–4 mg in 60 mL tap water) in 17 adults, resulting in clinical remission for 10 patients without adverse effects (without fatigue and headache and having normal electrolytes). This study highlighted the potential for user-friendly, home-based treatments for rectal disease. Finally, Jaeger et al. [41] conducted a retrospective analysis of TAC suppositories as an add-on therapy in 43 patients, with 60% achieving remission. In contrast to some of the earlier reports, this study also noted elevated trough levels (7.8 ± 2.5 μg/L) because of better absorption and one case of mild reversible renal impairment, underscoring the importance of monitoring for systemic absorption. Collectively, these studies demonstrate the efficacy of topical TAC in managing distal ulcerative colitis and refractory proctitis, with generally favourable safety profiles. However, they also highlight the need for careful monitoring, particularly when used as a long-term or add-on therapy.

**Table 2:** Studies focusing on the perirectal application of tacrolimus.

Settings	Study Type	Number of Patients	Interventions	Outcomes	Comments	Reference
Ulcerative proctitis	Pilot study	8	Rectal TAC ointment, 0.3 mg/mL	6/8 achieved remission; 5/8 reduced corticosteroids	8-week study	[39]
Refractory left-sided colitis or proctitis	Openlabel	19	TAC enema or suppository	13/19 showed improved disease activity	Low serum trough levels	[38]
Refractory ulcerative proctitis	RCT	21	Rectal TAC, 0.5 mg/mL vs. placebo	73% clinical response (TAC) vs. 10% (placebo)	8-week study	[42]
Refractory ulcerative proctitis	RCT	85	TAC suppositories, 2 mg vs. beclomethasone, 3 mg	63% clinical response in TAC group	4-week study	[43]
Refractory rectal disease	Openlabel	17	TAC enema, 1–4 mg in 60 mL tap water	Clinical remission in 10 patients	Up to 20-week study	[44]
Refractory IBD	Retrospective	43	TAC suppositories as add-on therapy	60% achieved remission	Elevated trough levels; 1 case of mild reversible renal impairment	[41]

TAC: tacrolimus; IBD: inflammatory bowel disease; RCT: randomised controlled trial.

## 7. Adverse Effects of Topical Tacrolimus

Topical TAC usually results in low (<3.2 ng/mL) or undetectable systemic levels, though some studies have found measurable serum concentrations. This highlights the need to monitor TAC levels regardless of the administration method, albeit with less concern than when TAC is given systemically [45]. The described adverse effects following the use of topical TAC are generally mild and localised, with burning or itching at the application site being the most common adverse effect [46]. Systemic side effects, such as nephrotoxicity and neurotoxicity, typically associated with oral TAC, are significantly lower or absent in most studies of TAC in the setting of IBD [47]. However, there are limited long-term safety data for topical TAC in IBD, emphasising the need for continuous vigilance and monitoring.

## 8. Innovative Delivery Systems for Topical Tacrolimus

Novel delivery systems may enable enhanced efficacy of topical TAC. As an example, Seoane-Viaño et al. [48] fabricated novel self-supporting TAC suppositories using semisolid extrusion three-dimensional (3D) printing. In a subsequent study, the same authors then evaluated the efficacy of these suppositories in a rodent model of colitis, with comparison to control animals that received no treatment [49]. The extent of wall thickening was assessed by imaging, and inflammatory status was evaluated histologically. Each of the outcome measures was improved 7 and 10 days after TAC treatment. This work demonstrated the efficacy of this mode of delivery but did not include comparison to another intervention or any other novel delivery systems.

Another different approach that may have future applications in the management of cutaneous manifestations of IBD is transdermal delivery with a blocking patch (BP). Zhao et al. [40] developed and validated this delivery method, focusing on its role in psoriasis. The investigators used TAC in a hyaluronic acid-based BP system and demonstrated transdermal release via confocal fluorescence microscopy. They then employed a murine model of psoriasis to compare TAC delivered by the BP to topical administration of the same drug and showed that the BP resulted in substantially greater efficacy. Although this promising work was focused on psoriasis, the potential value of this transdermal delivery method will likely also have relevance to cutaneous manifestations of IBD.

## 9. Limitations and Gaps of Knowledge

This review was limited to reports on topical tacrolimus published in English. The addition of reports in other languages might add to the description of topical TAC. Most of the available reports were retrospective, potentially affecting data quality and the comprehensive evaluation of primary outcomes or adverse events. Furthermore, most were open-label, of short duration, and included heterogeneous patient groups and outcome assessments. Few reports included biological outcomes such as faecal biomarkers or endoscopic follow-up. While some studies compared TAC to steroid treatments, comparative studies evaluating TAC against biologic therapies like infliximab were lacking. However, it is important to note that local administration of infliximab increases the likelihood of developing infliximab antibodies, a concern not associated with the local administration of calcineurin inhibitors such as TAC. This distinction highlights a potential advantage of topical TAC over locally administered biologics in terms of immunogenicity and long-term efficacy.

## 10. Conclusions

Topical TAC has been evaluated in several different groups of patients with IBD. Overall, the available (albeit limited) data suggest that topical TAC may have a role in the management of cutaneous features of CD and in the management of distal colitis. Furthermore, it appears that topical TAC is safe in these settings, with low concern about systemic effects. Further evaluations of TAC that include clear objective outcomes (including biological markers) and that compare outcomes to other currently available medications are required to more clearly substantiate the optimal role of topical TAC in individuals with IBD.

## Figures and Tables

**Table 1:** Reports of topical tacrolimus for cutaneous manifestations in individuals with IBD.

Settings	Study Type	Number of Patients	Interventions	Outcomes	Comments	Reference
Perioral and perineal CD in children	Observationl	9	TAC 0.5 mg/g ointment	7/8 improved after 6 weeks; complete healing in 1–6 months	Gradual tapering approach successful; undetectable serum TAC levels	[35]
Various cutaneous CD manifestations in adults	Observationl	20	TAC 0.1% ointment once daily for 12 weeks	15/17 improved; 1 complete resolution	Well tolerated; undetectable serum levels	[36]
Orofacial CD lesion	Case Report	1	TAC 0.1% ointment twice daily	Gradual reduction in lesion size over 12 months	Improved oral features	[37]

TAC: tacrolimus; CD: Crohn’s disease.
